# Efficacy Evaluation Study for Microburst Insulin Infusion: A Novel Model of Care

**DOI:** 10.3389/fpubh.2021.600906

**Published:** 2021-08-12

**Authors:** Steven W. Howard, Zidong Zhang, Jacob Linomaz, Wing Lam, Zhengmin Qian, Jerry Thurman, Rhonda BeLue

**Affiliations:** ^1^Department of Health Management and Policy, College for Public Health and Social Justice at Saint Louis University, St. Louis, MO, United States; ^2^Department of Epidemiology and Biostatistics, Saint Louis University, St. Louis, MO, United States; ^3^SSM Health Care, St. Louis, MO, United States

**Keywords:** insulin infusion, efficacy, neuropathy, diabetes, diabetic complications

## Abstract

**Objectives:** This study aims to evaluate the impact of Microburst Insulin Infusion (MII) treatment on Type 1 and 2 diabetic patients' HbA1c, lipids, peripheral neuropathy, and patient-reported health status.

**Methods:** We reviewed clinical charts, including lab results, for more than 80 diabetic and pre-diabetic patients treated at one U.S. outpatient clinic in St. Louis, Missouri between February 2017 and December 2019. Data included patient demographics, treatment data, lab and neuropathy tests, and self-reported patient health status questions.

The explanatory variable was number of months of MII treatment. Treatments are 3–4 h in length, with two intensive infusions the first week and one treatment each week thereafter, usually for 12 weeks total. Lab tests were at 12-week intervals.

Generalized linear modeling and t-tests assessed the significance of differences between patients' baseline lab values, neuropathy measures, and health status before treatment vs. after final treatment.

**Results:** Number of MII treatments per patient ranged from 1 to 262, over 1–24 months. Time in MII treatment was significantly associated with reductions in HbA1c by nearly 0.04 points per month, and triglycerides declined 3 points per month. Neuropathy measures of large toe vibratory sensation (clanging tuning fork) improved significantly, as did patient-reported health and feelings of improvement since beginning treatment.

**Discussion:** The MII therapy appears to be efficacious in treating diabetic patients, particularly those with complications like neuropathy. Our findings affirmed several other studies. We uniquely incorporated patient health questionnaires, and empirically studied MII treatment efficacy for diabetes in a population large enough to permit statistically valid inferences. With multiple waves of data for over 80 patients, this is one of the most extensive quantitative studies of microburst insulin infusion therapy conducted to date, with protocols more uniformly implemented and survey instruments more consistently administered by the same clinical team. Given the advances in insulin infusion therapy brought by MII, and early indications of its efficacy, the time is right for more in-depth studies of the outcomes patients can achieve, the physiological mechanisms by which they occur, MII's comparative effectiveness vis-à-vis traditional treatments, and cost-effectiveness.

## Introduction

Diabetes mellitus is one of the most prevalent chronic diseases, affecting nearly 10% of the world's population, and imposing a cost of nearly $1 trillion USD globally (1–3). This chronic health condition is characterized by hyperglycemia which results from defects in glucose homeostasis stemming from relative or absolute deficiencies in insulin. Type 2 diabetes, comprising 90–95% of all diabetics, stems from a relative insulin deficiency as a result of the body becoming increasing resistant to the glucose lowering effects of insulin. Between 5 and 10% of diabetics are type 1, characterized by an autoimmune process with genetic predispositions and potential environmental factors that results in destruction of insulin-producing Beta cells in the pancreas (4). Complications from both Type 1 and Type 2 diabetes are an increasing public health challenge, and the prevalence of conditions like neuropathy can be as high as 50% among diabetics (5). Most research and clinical interventions have focused on managing blood sugar and/or insulin to healthy levels, which delays the onset of complications or slows their advance. Few interventions have been effective in improving the body's production or utilization of insulin. For patients in the outpatient environment, subcutaneous insulin injection is the most common management approach for insulin-resistant patients. In the inpatient environment, continuous insulin infusion may be used. While these interventions have helped slow the decline in the health of diabetics, they have not been able to halt or reverse complications such as neuropathy. In this exploratory study, we begin to evaluate the hypothesis that Microburst Insulin Infusion (MII) may be efficacious in treating HbA1c and complications of diabetes.

Limited research exists on the efficacy of MII, the pulsatile mode of insulin delivery most similar to that naturally occurring in the body. Earlier forms of insulin infusion included outpatient intravenous insulin therapy (OIVIT), pulsatile intravenous insulin therapy (PIVIT), hepatic activation therapy (HAT), chronic intermittent intravenous insulin infusion (CIIIT), metabolic activation therapy (MAT), and the Harvard Protocol. These techniques pulsed insulin via intravenous infusion, but at increasing levels that progressively built up insulin levels in the body. If the magnitude of these insulin pulses is reduced or absent there can be a defect in hepatic intracellular signaling resulting in increased hepatic output of glucose and reduced glucose utilization in animal models resulting in onset of Type 2 diabetes (6). In an animal study, researchers evaluated three different insulin delivery methods. One subgroup received pulsatile insulin (PII), one received a continuous insulin infusion while the last had reduced amplitude pulsatile insulin as seen in Type 2 diabetes. They found that those that received PII had improved insulin action, while those who received a stable insulin infusion or reduced amplitude pulses of insulin (as seen in Type 2 Diabetes) had abnormal liver metabolism resulting in increased liver production of glucose and worsening hyperglycemia with insulin resistance. Previous researchers further postulated the abnormal liver response may be contributing to some complications of diabetes such as abnormal lipid metabolism and cardiovascular disease (7). Other researchers have had similar findings in human studies, supporting the potential for MII to produce superior blood glucose management compared to continuous insulin infusion (8, 9).

MII is a newer mode of delivery and works differently. It aims to more closely simulate the function of a healthy pancreas, which delivers insulin from the pancreatic Beta cell to the circulation in a pulsatile fashion that varies with glucose levels in the bloodstream. MII aims to coordinate the timing and pulsed levels/quantities of insulin secretion, mimicking the workings of a healthy pancreas. These pulses are in approximate 5–6 min intervals and adjust insulin dosage to the glucose levels in the patient's blood (10, 11).

The literature includes early human studies of PII, in its various stages of evolution from PIVIT to MII. To mimic normal insulin delivery to the body, early researchers investigated patients with Type 1 diabetes mellitus on multiple daily injections of insulin and poor control. These patients were given 7–10 pulses of intravenous insulin over an hour while ingesting carbohydrates in the form of glucose. These sessions were given three times per day. After 2 days of treatments, therapy was then 1 day per week (12, 13). The literature contains more detailed explanation of the MII protocol and the mechanism by which it functions (10, 11). In a systematic review article by Dong et al. (10), the authors concluded that intravenous pulsatile insulin therapy in diabetics can lead to normal liver insulin levels as seen in non-diabetic individuals, and improvements in peripheral neuropathy symptoms (10). The liver in a Type 2 diabetic relies on metabolism of lipids over that of carbohydrates. It was also determined that pulsatile insulin therapy can change hepatic glucose metabolism favoring carbohydrate metabolism over lipid metabolism, thereby reducing free fatty acids which can promote inflammatory responses elsewhere (11). Given the findings of this research and the experiences of clinicians treating diabetics using MII, we purposed to study the efficacy of MII in controlling blood sugar, lipids, peripheral neuropathy, and patient-reported health status.

## Methods

### Hypotheses and Methodological Strategy

We hypothesize that the longer a patient is treated with MII, the more their HbA1c, triglycerides, neuropathy, and self-reported health and wellbeing will improve, compared to the baseline of their first patient encounter. This is a retrospective cohort study delineating trajectories in each subject of laboratory and clinical measures from baseline. Should statistically significant improvement be observed for MII patients, a subsequent controlled trial may be in order.

### Data

We reviewed clinical charts, including lab results, for all diabetic and pre-diabetic patients treated at one outpatient clinic in St. Louis, Missouri, USA between February 2017 and December 2019. There were 86 patients in total (**Table 3A**), of whom 60 had lab data (of whom **Table 2** shows 52–56 had lab result data for each of the five outcome measures studied).

The explanatory variable of interest was the number of months (continuous) that patients were treated. The MII treatment sessions are ~3–4 h in length. The patient receives two intensive intravenous infusions the first week of treatment (typically 1–2 days apart), and one treatment each week thereafter, usually for 12 weeks total. Some patients choose to continue receiving MII treatments longer term, though frequency may reduce to bi-weekly or monthly. Lab tests were drawn at ~12-week intervals (a baseline panel of labs pre-treatment, another lab panel at week-12, and every 12 weeks thereafter for patients who opted to continue treatment).

The data abstracted from patient charts and questionnaires included lab values [HbA1c, total cholesterol, low-density lipids (LDL), triglycerides, and estimated glomerular filtration rate (eGFR)—all coded as continuous variables], patient demographics (age, sex, race, BMI, and diabetes type—all categorical variables), the duration of vibratory sensation from the CTF test (in seconds—continuous), and patient-reported health questions (0–10, 1–10, or percentage change—all coded as continuous). Each patient had between 1 and 262 MII treatments. At each treatment, a Family Nurse Practitioner (NP) administered the health questionnaire and clanging tuning-fork test (CTF) to assess neuropathy (14). The descriptive statistics are reported in **Table 3B**.

With the questionnaire, the NP asked each patient a series of standard self-reported health questions, coding their responses on the commonly-used 0–10 or 1–10 scale (i.e., “How is your diabetes-related pain on a scale of 1–10?”). The same Nurse Practitioner conducted all the patient questionnaires, and also recorded the duration of vibratory sensation from the big toe of each foot using a 128 Hz tuning fork (with a 15-s maximum/top-coding).

The questionnaire asked patients:

- How are you feeling today? (1–10 scale)
- How is your overall health? (1–10 scale)
- How is your diabetes-related level of pain? (1–10 scale)
- How does your diabetes-related pain interfere with your activities of daily living (ADLs)? (1–10 scale)
- How has your physical activity level changed since your last treatment? (% change)
- How has your energy level changed since your last treatment? (% change)
- How has your neuropathy changed since your last treatment? (% change)
- How has your sleep quality (including sleep pattern) changed since your last treatment? (% change)
- How has your vision changed since your last treatment? (% change)
- To what degree has your overall health changed since your FIRST treatment? (% change)

All the aforementioned measures were assessed at baseline and at each treatment session, and entered by the NP on the patient intake form. All data were subsequently abstracted into the analytic data set.

### IRB Approval

The Institutional Review Board of the academic institution determined that ethical approval for this study was not required in accordance with local legislation and national guidelines, as no individually identifying information was available to the research team.

### Statistical Modeling

Separate ordinary least squares regression models were run for the change in each outcome of interest (HbA1c, LDL cholesterol, triglycerides, total cholesterol, eGFR, vibratory sensation, and multiple self-reported health questions), comparing each patient's value at time of final (or most recent) treatment vs. the patient's baseline measures. Patient encounters that were missing data were omitted from the regression, and **Table 2** includes the number of patients with complete data included in each model (out of a total of 60 patients with lab results). Due to the smaller number of subjects, we used *t*-tests to gauge the significance of differences between patients' continuous self-reported, subjective outcome measures before treatment vs. after final (or most recent) treatment. To test for non-linear relationships, we produced a scatter plot of HbA1c by weeks of MII treatment (Supplementary Figure 1). We also performed *t*-tests on the differences in self-reported, subjective measures between the baseline and the final treatments, and further stratified the statistics based on the categories of patients who only completed 1–5, 6–10, 11–15, or >15 treatments (**Table 4**). We used SAS University Edition version 2.8, to generate separate generalized linear models (GLM command) to identify statistically significant differences for each outcome variable between patients' first treatment and final treatment.

## Results: Lab Test Data

Descriptive statistics for the Lab Test analyses are presented in Tables 1A,B. Most patients were age 45–64 (56.7%), though 15% were younger than 45, and 28.3% older than 64. Sixty percent were female. All but 4 were white (93.3%). Three were prediabetic, 26 Type 1, and 31 Type 2 diabetics. With respect to weight, 26.7% were normal or overweight, 40% were mildly obese, and 33.3% were severely obese.

**Table 1A:** Baseline descriptive statistics—lab data (categorical variables).

**Variable**	***N* (%) at baseline**
**Sex**	
Male	24 (40.0%)
Female	36 (60.0%)
**Race**	
White	56 (93.3%)
Black/African–American	4 (6.7%)
**Age (at first encounter)**	
<45 years	9 (15.0%)
45–64 years	34 (56.7%)
>65 years	17 (28.3%)
**BMI category**	
Normal or overweight	16 (26.7%)
Mild obesity	24 (40.0%)
Severe obesity	20 (33.3%)
**Diabetes type**	
Type 1	26 (43.3%)
Type 2	31 (51.7%)
Pre-diabetic	3 (5.0%)
**HbA1c**	
Well-controlled (<7.0)	16 (28.5%)
Fair-control (>7.0, <9.0)	24 (42.9%)
Poor-control (>9.0)	16 (28.5%)

**Table 1B:** Baseline descriptive statistics, continuous variables.

**Variable**	**Mean**	**Minimum, Maximum**
HbA1c	7.6	5.6, 10.9
eGFR	87.4	19, 173
LDL cholesterol	98.9	39, 170
Triglycerides	240.1	72, 617
Total cholesterol	170.4	106, 258

HbA1c measures ranged from 5.6 to 10.9 with a mean of 7.6 (Table 1B) (the CDC defines A1c levels below 5.7 as normal, 5.7–6.4 as pre-diabetic, and >6.4 as diabetic) 28.5% (16 patients) had well-controlled HbA1c; while 42.9% (24 patients) were moderately controlled (7.0 ≤ A1c <9.0); and 28.5% (16 patients) had poorly controlled A1c (≥9.0) (Table 1A). Estimated glomerular filtration rates (eGFR) ranged from 19 mL/min/1.73 m^2^ to 173 mL/min/1.73 m^2^ with a mean of 87.4 mL/min/1.73 m^2^ (the CDC defines eGFR levels >90 ml/min as normal, 30–90 as mild or moderate reductions in kidney function, and <30 ml/min as severe reduction in kidney function) LDL ranged from 39 to 170 mg/dLwith a mean of 98.9 mg/dL (the CDC defines LDL levels <100 mg/dL as normal) Triglycerides ranged from 72 to 617 mg/dL with a mean of 240.1 mg/dL (the CDC defines Triglyceride levels <150 mg/dL as normal) Total cholesterol ranged from 106 to 258 mg/dL with a mean of 170.4 mg/dL (the CDC defines Total Cholesterol levels <200 mg/dL as normal).

The results of each model for the Lab Test data are presented in Table 2. The primary explanatory variable of interest, the time variable for months of MII treatment, was associated with reductions in HbA1c levels by 0.038 A1c points per month, with a *p*-value of 0.067. This supported the hypothesis that MII can reduce triglycerides (*p* = 0.035), and HbA1c (*p* = 0.067) (Having poorly controlled HbA1c at baseline was also associated with reducing HbA1c). None of the other covariates had statistically significant associations in any of the models (Black/African–American race appears to be associated with lower LDL and Total Cholesterol levels, but there were only three African-Americans in the data). The significance of the F-statistic and magnitude of *R*^2^ coefficient indicate the model is a strong predictor of reduced HbA1c among the diabetic patients in the study.

**Table 2:** Regression models—lab data (changes in outcome measures).

**Variable**	**HbA1c β** **(p-value)**	**LDL β** **(p-value)**	**Triglyceride β** **(p-value)**	**Total Cholest-erol β** **(p-value)**	**eGFR β** **(p-value)**
Months in MII treatment	−0.038 (***p*** **=** **0.067**)	−0.291 (*p* = 0.534)	−3.115 (***p*** **=** **0.035**)	−0.440 (*p* = 0.390)	−0.045 (*p* = 0.801)
Male gender	−0.230 (*p* = 0.558)	−14.353 (*p* = 0.079)	62.776 (***p*** **=** **0.013**)	2.100 (*p* = 0.809)	−2.375 (*p* = 0.441)
Black/African American	−0.679 (*p* = 0.397)	−40.762 (***p*** **=** **0.020**)	9.659 (*p* = 0.858)	−42.617 (***p*** **=** **0.029**)	11.232 (*p* = 0.098)
**Age (at first encounter)**					
<45 years (vs. age 45–64)	0.061 (*p* = 0.886)	4.969 (*p* = 0.611)	−49.157 (*p* = 0.102)	3.038 (*p* = 0.772)	−6.401 (*p* = 0.086)
>65 years (vs. age 45–64)	−0.129 (*p* = 0.795)	2.873 (*p* = 0.790)	−35.029 (*p* = 0.308)	2.957 (*p* = 0.807)	−5.552 (*p* = 0.198)
**BMI category**					
Mild obesity (vs. normal/overweight)	−0.460 (*p* = 0.259)	−7.07 (*p* = 0.444)	2.689 (*p* = 0.925)	−2.743 (*p* = 0.787)	−0.672 (*p* = 0.853)
Severe obesity (vs. normal/overweight)	−0.136 (*p* = 0.757)	−1.178 (*p* = 0.906)	14.180 (*p* = 0.648)	−2.323 (*p* = 0.832)	−6.349 (*p* = 0.113)
**HbA1c control category**					
Fair control (vs. good control)	−0.033 (*p* = 0.939)	N/A	N/A	N/A	N/A
Poor control (vs. good control)	−1.299 (***p*** **=** **0.017**)	N/A	N/A	N/A	N/A
Model *R*^2^ goodness of fit statistic	0.371	0.188	0.238	0.143	0.236
Model *F*-statistic and *p*-value	3.02 (***p*** **=** **0.007**)	1.46 (*p* = 0.208)	2.10 (*p* = 0.062)	1.12 (*p* = 0.368)	1.99 (*p* = 0.078)
Number of patients w/usable data	56	52	55	55	53

*Bold p-values indicate statistically significant coefficients*.

## Results: Neuropathy and Patient-Reported Data

Separately, descriptive statistics for the Neuropathy and Patient-Reported Data Set (hereafter referred to as “Subjective Data”) are presented in Tables 3A,B. Twenty-six patients had these data in their charts, but no lab results data. Consequently, the Subjective Data had 86 patients, whereas the lab data had only 60. Patient ages ranged from 19 to 85 with a mean of 57. Fifty-two were female and 34 were male. All but five were white. Four were prediabetic and averaged 11 treatments per patient during the study period. Fifty-three were Type 1 and averaged 28.6 treatments per patient, and 29 were Type 2 diabetics and averaged 24.5 treatments each. Number of MII treatments per patient ranged from 1 to 262.

**Table 3A:** Descriptive statistics—self-report/subjective data (categorical variables).

**Variable**	***N* (%)**
**Sex**	
Male	34 (39.5%)
Female	52 (60.5%)
**Race**	
White	81 (94.2%)
Black/African-American	5 (5.8%)
**Age (at first encounter)**	
<45 years	11 (12.8%)
45 to 64 years	47 (54.7%)
≥65 years	27 (31.4%)
**BMI category**	
Normal or overweight	16 (18.6%)
Mild obesity	24 (27.9%)
Severe obesity	19 (22.1%)
**Diabetes type**	
Type 1	53 (61.6%)
Type 2	29 (33.7%)
Pre-diabetic	4 (4.6%)
**HbA1c**	
Well-controlled (<7.0)	16 (18.6%)
Fair-control (≥7.0, <9.0)	22 (25.6%)
Poor-control (>9.0)	15 (17.4%)
(missing data)	33 (38.4%)

**Table 3B:** Descriptive statistics—self-report/subjective data (continuous variables).

**Variable**	**Mean**	**Minimum, Maximum**
Age (years)	57	19, 85
Total # of MII treatments per patient	24.7	1, 262
How are you feeling?	7.29	1, 10
How is your overall health?	7.22	1, 10
How is your diabetes-related pain?	4.80	1, 10
Diabetes pain interference w/ ADLs?	4.72	1, 10
Vibratory sensation—right foot (seconds)	9.37 s	0, 15
Vibratory sensation—left foot (seconds)	9.13 s	0, 15
Overall improvement since starting MII?	47.2%	−25, 120%
Physical activity change? (since last treatment)	33.4%	−65, 200%
Energy change? (since last treatment)	34.5%	−65, 100%
Neuropathy change? (since last treatment)	38.7%	−40, 100%
Sleep pattern/quality change? (since last treatment)	39.4%	−50, 100%
Vision change? (since last treatment)	24.2%	−10, 100%

Patients were asked to self-report on 10 health status questions, and the clinic NP measured vibratory sensation in both feet using a 128 Hz clanging tuning fork (CTF). Patients reported a mean of 9.1 and 9.4 s of vibratory sensation in the left and right feet, respectively, with a range of 0–15 s. Patients reported averages of 7.29 and 7.22 on the “How are you feeling?” and “How is your overall health?” questions, respectively, with ranges of 1–10. On the “How is your diabetes-related level of pain?” and “How is that pain interfering with your Activities of Daily Living (ADLs)?” questions, patients' average responses were 4.8 and 4.72, respectively, with ranges of 0–10. Patients were asked to rate their overall percentage health improvement since beginning treatment. Assessed at each treatment encounter, responses indicated a 47.2% improvement on average, ranging from −25% to 120%. The final five patient self-reported measures and their mean percentage changes were: physical activity change (33.4%), energy change (34.5%), neuropathy change (38.7%), changes in sleep patterns or sleep quality (39.4%), and vision change (24.2%).

The results of the *t*-tests are presented below in Table 4, including pre-/post-treatment differences and the *p*-values of their differences to indicate statistical significance (*p* < 0.05 indicates significant differences).

**Table 4:** *T*-tests: significance of pre-/post-differences in outcome measures—patient-reported/subjective data.

**Variable**	**Overall pre-/post: last visit vs. baseline**	**Categorized: 1–5 visits vs. baseline**	**Categorized: 6-10 visits vs. baseline**	**Categorized: 11–15 visits vs. baseline**	**Categorized: 16–30 visits vs. baseline**
Vibratory sensation—right foot (seconds)	2.99 (***p*** **<** **0.001**)	2.67	1.86	1.27	4.32
Vibratory sensation—left foot (seconds)	3.46 (***p*** **<** **0.001**)	3.67	1.29	2.43	4.00
How are you feeling?	0.54 (***p*** **=** **0.011**)	−0.45	1.18	0.50	0.79
How is your overall health?	0.69 (***p*** **<** **0.001**)	−0.34	0.18	0.67	1.29
How is your diabetes-related pain?	−0.52 (*p* = 0.131)	0.00	−0.85	0.33	−0.33
Diabetes pain interference w/ADLs?	0.01 (*p* = 0.977)	−1.00	−0.70	0.57	−1.05
Overall improvement since starting MII?^*^	25.38% (***p*** **<** **0.001**)	7.17%	6.15%	23.91%	38.88%
Physical activity change? (since last treatment)	NS	NS	NS	NS	NS
Energy change? (since last treatment)	NS	NS	NS	NS	NS
Neuropathy change? (since last treatment)	NS	NS	NS	NS	NS
Sleep pattern/quality change? (since last treatment)	NS	NS	NS	NS	NS
Vision change? (since last treatment)	NS	NS	NS	NS	NS

* *MII, Microburst Insulin Infusion therapy. Bold p-values indicate statistically significant coefficients*.

Statistically significant improvements of 3–3.5 s were observed for the CTF vibratory sensation for the right and left feet, respectively. Patient self-reports of feeling better and experiencing improved overall health, 0.54 and 0.69, respectively (on a scale of 1–10), were also statistically significant. Lastly, patients reported a statistically significant improvement, >25%, since beginning the MII treatment.

## Discussion

We aimed to study relationships between patients' number of weeks in MII treatment (changes between baseline and last or most recent treatment) and their associated HbA1c, triglycerides, neuropathy symptoms, and self-reported health. Overall, patients experienced improvements in both lab values and self-reported measures the longer they were in treatment. Table 4 showed short-term reductions in self-reported health scores, and the NP and clinic medical director posited that patients' reports of how they are feeling, diabetic-related pain and overall health may worsen in the short-run as neuropathy symptoms diminish, sensation returns, and they begin to experience pain again. Our generalized linear models show HbA1c declining approximately 0.038 A1c points per month, and triglyceride levels declining ~3 mg/dl per month in MII therapy. This finding is in line with, and potentially higher than the ~4–12% reductions observed over 1–4 years in other studies, including results from studies of metformin or metformin plus additional drugs (15), sulphonylureas (16), TZD drugs (17), and inhaled insulin (18). Vibratory sensation measures also improved, as did patients' self-reported measures of how they were feeling, and their overall health also improved at statistically significant levels. These findings are also in line with results published over the past 10–20 years, including those of Elliott et al. (11) (8–13, 19–23).

### Previous Research

Our findings were consistent with some of the earlier research on PII generally, or MII more specifically. Multiple studies supported the hypothesis that pulsed insulin infusions could be more efficacious than continuous insulin infusion (8, 9, 20–22). Our results were also consistent with the findings of the systematic literature review by Dong et al. (10) and Elliott et al. (11), we also found improvements in neuropathy (10, 11). However, most studies of MII have focused on metabolic measures estimated from patients' respiratory O_2_ and CO_2_ (10, 11). We focused on the lipid, HbA1c, eGFR, neuropathy measures, and patient self-reported health screening questions more commonly used in ambulatory care clinics globally. Our study is differentiated by incorporating the differences between MII and earlier pulsatile insulin infusion approaches, longer period of study (up to 3 years), a relatively large sample size, stronger methodology, and focus on clinical measures more commonly used by clinicians treating diabetics. Taken together, these differentiating factors make the current research an important addition to the body of literature on diabetes care.

### Strengths

This is one of the few studies to empirically study the efficacy of the MII treatment for diabetes in a population large enough to permit statistically valid inferences. With multiple waves of data on over 80 patients, this is one of the most extensive quantitative studies of microburst insulin infusion therapy conducted to date, with protocols more uniformly implemented and survey instruments more consistently administered by the same clinical team. Consistent with our hypotheses, we found statistically significant improvements in HbA1c, triglycerides, neuropathy, and 3 self-reported patient survey measures of health and well-being, supporting the hypothesis that the MII therapy is efficacious in treating diabetic patients, particularly those with complications like neuropathy. This study is also one of the few to implement surveys of patients' self-reported health and quality of life. Like the findings of Dong et al. (10) and Elliott et al. (11), patients reported significantly improved health after the 12-week course of treatment, and frequently even longer-term.

### Limitations

While this study is pioneering, it has inherent limitations. Our hypothesis was that MII would show early indications of potential efficacy, using solely existing chart data for patients served from February 2017 through December 2019. Given frequent contact with patients over time, the Hawthorne effect and regression to the mean may have impacted results. In the current retrospective cohort study context, we were not able to include a control group, and the *t*-tests and regression models effectively used subjects as their own controls over weeks of time in treatment. Sample size is also a concern. While this is one of the largest studies yet conducted on the MII treatment, statistical power was reduced, limiting our options for statistical modeling. This may also have contributed to lack of statistical significance of the findings for low-density lipids (LDL cholesterol) and estimated glomerular filtration rate (eGFR), measures we expected to improve with time in treatment.

The CDC estimates over 30 million Americans have diabetes, and the WHO estimated the global count at over 400 million in 2014 and identify the disease as a major cause of heart disease, stroke, blindness, and amputations (2, 3, 24, 25). In 2017, diabetes-related costs of care were ~$10,000 per diabetic patient, with the disease imposing billions of dollars in indirect costs just in the U.S. alone (25). Better managing the degenerative effects of diabetes would not only decrease pain and suffering of patients, but could also save trillions of dollars in direct and indirect costs worldwide.

Given the promising preliminary findings of this retrospective study, further research is warranted. The research team plans a second phase of the study to compare MII results observed to-date with results observed among a retrospective cohort of diabetic patients managed through traditional lifestyle modification (diet and exercise) treatment protocols at their affiliated academic medical center. If that study shows comparatively superior outcomes, a multi-center, prospective clinical trial should be pursued. Given the advances in insulin infusion therapy brought by MII, and early indications of its efficacy, the time is right for more in-depth studies of the outcomes patients can achieve, the physiological mechanisms by which they occur, MII's comparative effectiveness vis-à-vis traditional treatments, and cost-effectiveness.

## Data Availability Statement

The raw data supporting the conclusions of this article will be made available, subject to approval by the clinic leadership.

## Ethics Statement

The studies involving human participants were reviewed and approved by the Saint Louis University Institutional Review Board. Written informed consent for participation was not required for this study in accordance with the national legislation and the institutional requirements. Data were provided de-identified to the researchers, and the study was deemed exempt.

## Author Contributions

SH and ZZ managed the entirety of the study. JL and WL provided valuable assistance with literature review, data entry, and writing. ZQ, JT, and RB provided important subject matter consultation, advice on study design, and contributed to the writing process. All authors contributed to the article and approved the submitted version.

## Conflict of Interest

SH previously consulted on a clinical improvement and strategic planning project with Mitokon Health Center, the St. Louis clinic from which the de-identified data were obtained. The remaining authors declare that the research was conducted in the absence of any commercial or financial relationships that could be construed as a potential conflict of interest.

## Publisher's Note

All claims expressed in this article are solely those of the authors and do not necessarily represent those of their affiliated organizations, or those of the publisher, the editors and the reviewers. Any product that may be evaluated in this article, or claim that may be made by its manufacturer, is not guaranteed or endorsed by the publisher.
