# Supplementary material for: Efficacy Evaluation Study for Microburst Insulin Infusion: A Novel Model of Care
Source: Front Public Health. 2021 Aug 12;9:600906. doi: 10.3389/fpubh.2021.600906 (PMC8387654; doi:10.3389/fpubh.2021.600906)
Supplement: Supplementary file 1 [file Table_1.docx]

**Supplementary Figure 1**. Scatter plots of HbA1c change between the first and last encounters.


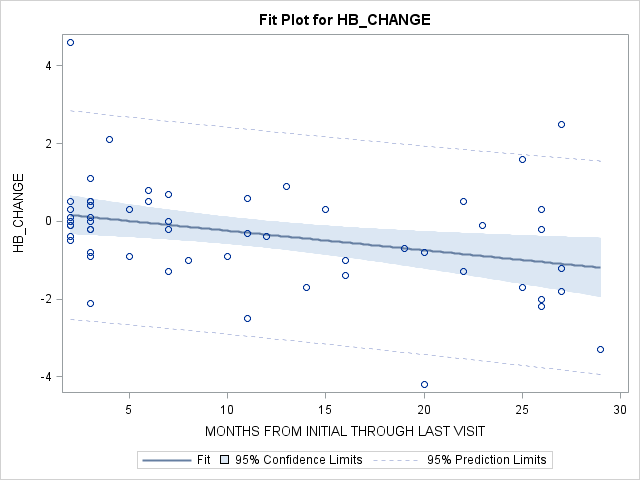


Note: the “HB_CHANGE” variable is change from subjects’ first and final/most recent HbA1c measures.
